# Analysis of cd45- [cd34+/kdr+] Endothelial Progenitor Cells as Juvenile Protective Factors in a Rat Model of Ischemic-Hemorrhagic Stroke

**DOI:** 10.1371/journal.pone.0055222

**Published:** 2013-01-31

**Authors:** Julius L. Decano, Ann Marie Moran, Nicholas Giordano, Nelson Ruiz-Opazo, Victoria L. M. Herrera

**Affiliations:** Department of Medicine and Whitaker Cardiovascular Institute, Boston University School of Medicine, Boston, Massachusetts, United States of America; University of Queensland, Australia

## Abstract

**Background:**

Identification of juvenile protective factors (JPFs) which are altered with age and contribute to adult-onset diseases could identify novel pathways for reversing the effects of age, an accepted non-modifiable risk factor to adult-onset diseases. Since endothelial progenitor cells (EPCs) have been observed to be altered in stroke, hypertension and hypercholesterolemia, said EPCs are candidate JPFs for adult-onset stroke. *A priori*, if EPC aging plays a ‘master-switch JPF-role’ in stroke pathogenesis, juvenile EPC therapy alone should delay stroke-onset. Using a hypertensive, transgenic-hyperlipidemic rat model of spontaneous ischemic-hemorrhagic stroke, spTg25, we tested the hypothesis that freshly isolated juvenile EPCs are JPFs that can attenuate stroke progression and delay stroke onset.

**Methodology/Principal Findings:**

FACS analysis revealed that cd45- [cd34+/kdr+] EPCs decrease with progression to stroke in spTg25 rats, exhibit differential expression of the dual endodthelin-1/VEGFsp receptor (DEspR) and undergo differential DEspR-subtype specific changes in number and in vitro angiogenic tube-incorporation. In vivo EPC infusion of male, juvenile non-expanded cd45-[cd34+/kdr+] EPCs into female stroke-prone rats prior to stroke attenuated progression and delayed stroke onset (*P*<0.003). Detection of Y-chromosome DNA in brain microvessels of EPC-treated female spTg25 rats indicates integration of male EPCs into female rat brain microvessels. Gradient-echo MRI showed delay of ischemic-hemorrhagic lesions in EPC-treated rats. Real-time RT-PCR pathway-specific array-analysis revealed age-associated gene expression changes in cd45-[cd34+/kdr]EPC subtypes, which were accelerated in stroke-prone rats. Pro-angiogenic genes implicated in intimal hyperplasia were increased in stroke-prone rat EPCs (P<0.0001), suggesting a maladaptive endothelial repair system which acts like a double-edged sword repairing while predisposing to age-associated intimal hyperplasia.

**Conclusions/Significance:**

Altogether, the data demonstrate that cd45-[cd34/kdr+]EPCs are juvenile protective factors for ischemic hemorrhagic stroke as modeled in the spTg25-rat model. The ability to delay stroke onset emphasizes the importance of EPC-mediated roles in vascular health for ischemic-hemorrhagic stroke, a high unmet need.

## Introduction

Although much research has focused on environmental risk factors for adult-onset diseases as a paradigm for prevention and intervention, the counterpart concept of “juvenile protective factors” is less studied but equally important. As a conceptual construct, juvenile protective factors (JPFs) are endogenous physiological factors – be it cells, proteins, nucleic acids, or biochemical equivalent – which prevent disease in the young but which undergo age-associated cumulative changes which contribute to adult-onset diseases. *A priori*, targeting the maintenance of juvenile protective factors comprises a mechanism-based paradigm for the prevention and intervention of diseases associated with aging, such as stroke, neurodegenerative and/or cardiovascular diseases. In this way, instead of aging being a non-modifiable risk factor, JPFs become a translational pathway to modifying the contribution of aging to disease pathogenesis.

As part of the endothelial repair mechanism, endothelial progenitor cells (EPCs) are *a priori* putative juvenile protective factors for vascular health. Although there are controversies regarding definition and methods of isolation, as a concept EPCs are circulating cells involved in endothelial repair, maintenance of capillary network, and postnatal vascularization and angiogenesis in both physiological (e.g., wound healing) and pathological (e.g., tumor) conditions [1]–[3]. Decreases in total or subtype-specific EPC numbers and EPC dysfunction have been observed and associated with poor outcomes in cardiovascular disease and stroke [3]–[5], although acute cardiac and stroke events can be associated with increased EPCs due to acute mobilization [6], thereby demonstrating dynamic complexity of EPC levels. The deduced critical roles of EPCs have prompted several clinical studies testing efficacy of EPC-therapy for cardiac function restoration after myocardial infarction, although with successful as well as non-successful results [2]–[5]. The observed disparate outcomes of EPC-therapies have been attributed to variations in EPC subtype characterization and isolation methods [2]–[6]. However, while methodological variations are contributory, the non-consensus in EPC terminology and divergent clinical study results could altogether represent a dynamic complexity in EPC biology involving subtypes still to be defined and non-linear subtype-specific modulation by risk factors and aging. In fact, with organ-specific vascular endothelial heterogeneity, corresponding repair mechanisms and hence EPC subtype-specific actions are to be expected [3].

Currently, most pre-clinical EPC studies focus on *ex vivo* culture-expanded EPCs that are cd45+ [cd34+/kdr+]EPCs since cd45- [cd34+/kdr+]EPCs do not undergo ex vivo expansion [1], and since only ex vivo expanded cd45+ [cd34+/kdr+]EPCs are pro-angiogenic in vitro and in vivo in contrast to freshly isolated cd45+ [cd34+/kdr+]EPCs which are not [1]. However, since *ex vivo* culture expansion is, *a priori* not a physiological paradigm, and since freshly isolated (non-expanded) cd45+ [cd34+/kdr+]EPCs have been observed to not induce revascularization [1], we hypothesize that circulating cd45- [cd34+/kdr+]EPCs underlie *in vivo* real-time endothelial repair for the maintenance of endothelial integrity and microvascular networks, and hence are critical juvenile protective factors for microvascular health. To test this, we focused on ischemic-hemorrhagic stroke because of the key role of the microcirculation in cerebral ischemia and hemorrhagic transformation.

Ischemic-hemorrhagic strokes and hemorrhagic transformation after an ischemic stroke are major causes of morbidity and mortality and remain without major treatment breakthroughs. Based on the successful modeling of the clinical spectrum of cerebral chronic low-flow ischemia, microvascular paucity, microhemorrhages and subsequent ischemic-hemorrhagic strokes in a genetically hypertensive/transgenic-hyperlipidemic rat model, spTg25 rat model [7], [8], we deduce that microvascular paucity leads to chronic low flow-ischemia which then predisposes the brain to ischemic-hemorrhagic transformation. Current stroke therapeutic approaches address patency and neuroprotection but not microvascular paucity and its resultant chronic low-flow ischemia. We therefore tested the translational corollary that preservation of microvascular health in old stroke-prone Tg25+ rats through juvenile EPC-mediated repair would attenuate the course of chronic low-flow ischemia, microvascular paucity, and subsequent increased risk for ischemic-hemorrhagic infarction in the spTg25 rat model, thus delaying stroke onset.

Here we report that EPC subtype-specific changes occur in a non-linear fashion in response to developmental programming of increased stroke susceptibility, that fresh cd45- [cd34+/kdr+]EPCs exhibit subset-specific functions and pathway-specific profile changes with age, and that cell-therapy with freshly isolated juvenile cd45- [cd34+/kdr+]EPCs delays the onset of ischemic-hemorrhagic strokes in the spTg25 rat model.

## Materials and Methods

### Set-up of Stroke-prone Rats for Study

All rat studies were done in strict accordance with the recommendations in the Guide for the Care and Use of Laboratory Animals of the National Institutes of Health. The protocol was approved by the Boston University School of Medicine Institutional Animal Care and Use Committee (IACUC Protocol Number AN14055). The inbred, transgenic[hCETP]25 Dahl Salt-sensitive rat line (Tg25) was maintained in-house as heterozygous in pathogen-free conditions, and stroke-prone Tg25+ rats were bred on Purina 5001 regular rat chow with 0.4% NaCl as described [7]. Tg25+ rats were developed by pronuclear microinjection of human cholesteryl ester transfer protein transgene resulting in combined hyperlipidemia with increased total plasma cholesterol and triglycerides on regular rat chow with greater hyperlipidemia in males than female rats, but greater stroke susceptibility in female rats [7], [8]. Littermate rats were used for study and randomly assigned for study groups ascertaining that litters were evenly split between control and treated study groups. Rats were monitored daily for stroke onset. Upon detection of stroke associated neurologic deficits such as seizures, paralysis, loss of consciousness, rats were euthanized and lifespan recorded. Two-month old male non-stroke prone, non-transgenic rats of the identical inbred background were used as donors for EPC-therapy infusions. Blood samples were taken under Ketamine/Xylazine anesthesia, and all efforts were made to minimize suffering.

### FACS Analysis of EPC Subtypes

To determine rat EPC subtypes by FACS analysis, we performed FACS analysis on the buffy coat from 1 ml of whole blood in citrate dextrose buffer from each rat per study group. Following standard FACS protocols Immunophenotyping was performed using fluorescently labeled primary antibodies: AF647-anti-rat KDR, AF488 anti-ratCD34 (Sta. Cruz Biotech, CA), and PE anti-ratCD45 (BD Biosciences, CA), and PerCP Cy5.5-anti-ratDEspR antibodies at 1 ug/ml. Cells were washed and fixed in Cytofix buffer and filtered (BD Biosciences, CA). Differential immunophenotyping-fluorescence intensities were read in a BD FACS scanner and analyzed using Summit version 4.0 (Dako, CA).

### Isolation of cd45- [cd34+/kdr+]EPCs for Infusion Therapy and Tube Incorporation Assays

In order to minimize “time-out-of-body” and processing steps, EPCs were isolated directly from the “buffy coat” mononuclear cell layer from 10-mls of rat whole blood in citrate phosphate dextrose buffer (Sigma, MO) after removal of contaminating red blood cells (rbc) using NH_4_Cl_2_ rbc lysis buffer for FACs analysis (BD Sciences). Immunophenotyping was performed using AF647-anti-rat KDR, AF488 anti-ratCD34 (Sta. Cruz Biotech, CA), and PE anti-ratCD45 (BD Biosciences, CA), and PerCP Cy5.5-anti-ratDEspR (dual endothelin1/VEGFsp receptor) antibodies at 1 ug/ml in 200 ul ice-cold staining buffer (BD Biosciences, CA) for 1 hour in the dark, followed by 2 washes in 2% FBS, 1×PBS solution. Cells were then resuspended in 500 ul wash buffer and cd45- [cd34+/kdr+]EPCs were isolated using MoFlo high-speed cell sorter (Beckman Coulter, CA) with gating for cd45-, cd34+ and kdr+ EPCs for EPC-infusion therapy experiments. For tube incorporation experiments and nano-RT2 pathway-specific PCR array analysis, anti-DEspR immunophenotyping was added, and the additional DEspR+ vs. DEspR- gating performed during MoFlo high-speed cell sorting. MoFlo high-speed cell sorting was done after parameters were adjusted using control beads, prepared single-color control samples, and a universal negative control. Gated EPCs were collected in 200 ul sterile phosphate buffered saline with 2% fetal bovine serum for EPC therapy or in endothelial complete medium for tube incorporation assays. For RNA isolation for array analysis, EPCs were immediately subjected to quick-freeze after collection.

### EPC Therapy Studies, Stroke Monitoring and 11.7 Tesla MRI

EPCs were isolated from 8-week old donor non-stroke prone male inbred-Dahl S rats on the day of infusion and infused immediately via tail vein into recipient inbred-Dahl S stroke prone Tg25+ rat females at 3-months and 4-months of age prior to any stroke signs. Vehicle 1×PBS was injected into controls at identical time points. Stroke onset was determined by the detection of neurologic deficits such as seizures, paresis, paralysis, athetoid movements. Ex vivo 11.7T MR-imaging was performed on brains from control and stroke-prone rats as described [7].

### DNA Isolation and Y-chromosome PCR-testing

To confirm EPC integration, another set of recipient spTg25+ female rats were infused with male donor EPCs and analyzed at 1-, 2- and 3-weeks after infusion to test for Y-DNA presence. Y-chromosome DNA testing was done on DNA isolated from kidney, half-brain and microvessels isolated from the other half-brain as described [9]. DNA was isolated using standard protocols from brain microvessels, brain, kidney tissues. After cleanup of genomic DNA samples using Qiagen’s DNeasy kit with on-column RNase digestion, PCR-testing for Y-chromosome DNA using primers as described [10]. Radiolabeled PCR products were size fractionated on a 6% denaturing polyacrylamide gel and the expected 104bp product documented radiographically. Male-rat DNA sample was used as positive control.

### EPC Angiogenesis Tube-incorporation Assays

Tube incorporation with human umbilical vein cells (HUVECs) undergoing tubulogenesis was analyzed using standard angiogenesis conditions with Matrigel basement matrix mix (BD Biosciences, CA). To distinguish EPCs from HUVECs, EPCs were labeled with pkh26 red fluorescent cell linker (Sigma, MO) after MoFlo high speed cell sorting isolation as described above. Isolated pkh26-labeled EPCs in 200 ul complete medium was mixed with 5,000 HUVECs in 300 µl of complete medium and then layered onto matrigel-coated culture slide system (ThermoFisher, MA). After 16–18 hours to allow tube formation, EPC-incorporation into HUVEC-tubes was viewed and photographed using a Nikon Epifluorescence microscope using identical exposures.

### RT2 Pathways Specific Nano-array Analysis

Total cellular RNA with no contaminating DNA was isolated from subtype-EPCs from 6 rats per study group. Per study group, EPC-RNA samples from 2 rats were pooled for a total of 3 independent biological replicates. Six arrays were used to obtain 2 technical replicates per RNA pool. Pathway-specific array analysis was done using RT2 nano-preAmp cDNA synthesis kit, RT2 Profiler PCR arrays, and Rat Pathway Finder array with angiogenesis, apoptosis, stem cell, and cell senescence pathways represented (SABiosciences, CA), and run on an ABI StepOnePlus instrument. Quality controls comprised of equivalent positive PCR and reverse transcription controls among samples, and absence of reverse transcription inhibitors and DNA contamination. Genes with cycle threshold (CT) values >35 were considered not-expressed as recommended. The average of CT values were obtained only when variation among the 6 replicates was <10%. Per gene, the ratio of gene expression between different test groups to a common reference group was calculated as delta CT, and the fold change calculated as 2^deltaCT^ where deltaCT = average CT of gene-X of test group – average CT of gene-X of reference group. Two-fold or greater vectorial change in gene expression between test groups were cataloged per pathway and analyzed for statistical significance.

### Statistical Analysis

Data were assessed for normality and presented as means ± sd. One-way ANOVA and Tukey’s multiple comparison tests were used to compare differences in EPC subtype-specific levels and functional parameters among different study groups. Kaplan Meier survival curve analysis and Holm-Sidak test for P values were used to determine efficacy of EPC-therapy. Two-way (gene×study group) ANOVA and Holm-Sidak multiple pairwise comparisons were done to assess statistically significant gene expression changes. GraphPad Prism (GraphPad, CA) and SigmaStat (SysStat, CA) software programs were used for statistical analyses.

## Results

To determine whether EPCs are juvenile protective factors for microvascular health, we performed a series of quantitative and qualitative analyses of the effects of age, sex and stroke susceptibility on freshly isolated, non-“culture-expanded” cd45- [cd34+/kdr+]EPCs. The focus on non-expanded EPCs is based on the logic that real time in vivo surveillance and repair of endothelial cell turnover or injury would require circulating “on-the-ready” EPCs to detect, home to, and repair endothelial turnover and injury, prior to platelet adhesion and thrombosis. Experiments were therefore designed to test whether cd45- [cd34+/kdr+]EPCs quantitatively and/or qualitatively decline with age, and test the converse whether juvenile EPCs contribute to “on-the-ready” real-time surveillance and endothelial repair critical for the maintenance of endothelial integrity, thus capable of delaying stroke onset.

### Subtype-specific Changes in Circulating EPCs

**Figure 1:**
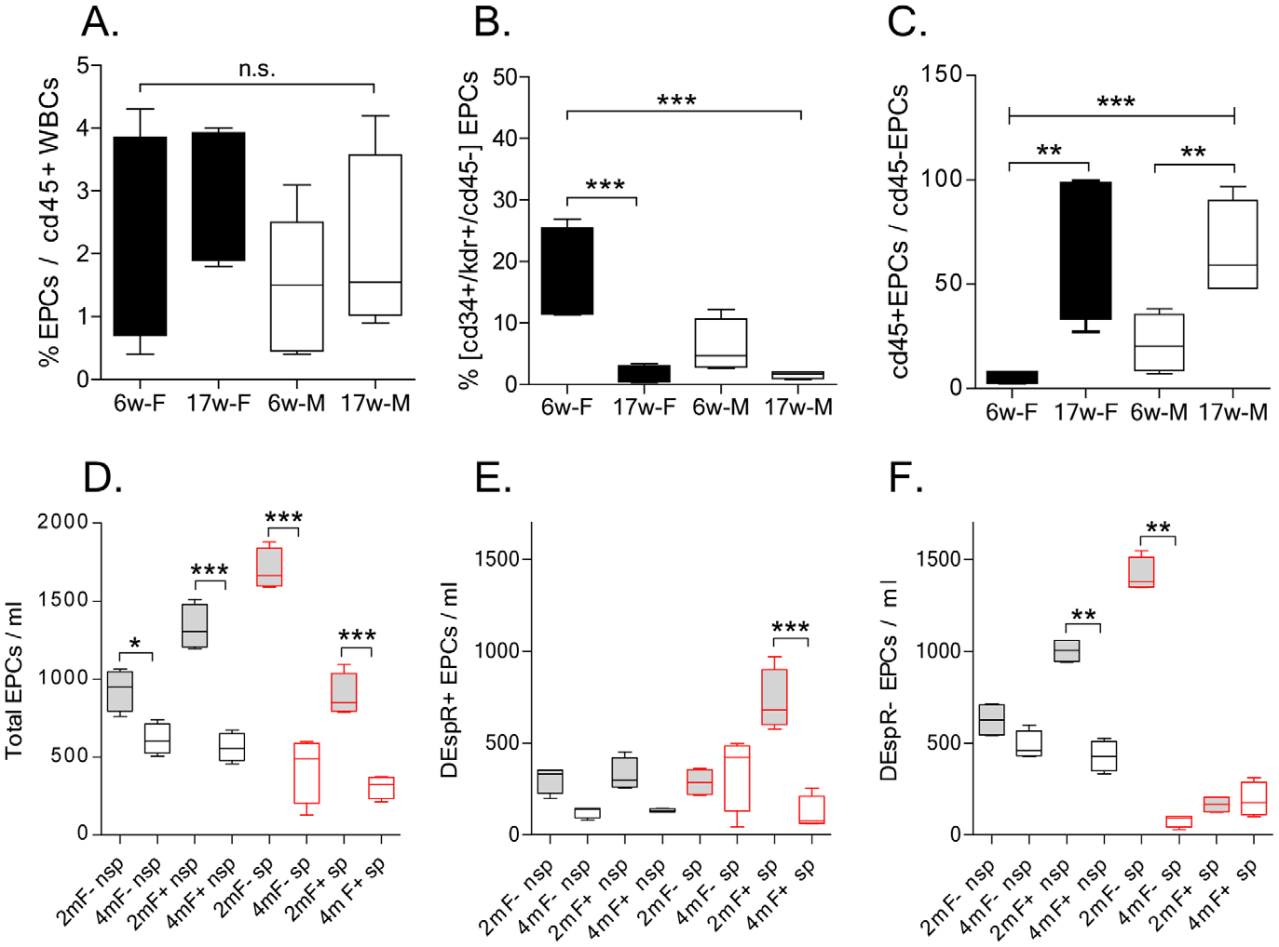
FACS analysis of age- and sex-specific changes in cd34+/kdr+ EPC subtypes. A) Analysis of the ratio of cd34+/kdr+ EPCs to cd45+ white blood cells as %, in males (M) compared to females (F) at 6-weeks (6w) and 17-weeks (17w) of age in non-stroke prone rats. B) Analysis of the ratio of cd45-[cd34+/kdr+]EPCs to total [cd34+/kdr+]EPCs as %, and C) analysis of the ratio of cd45+ to cd45- [cd34+/kdr+]EPCS in male and female rats at 6- and 17-weeks of age in non-stroke prone rats shown in A. D) Analysis of cd45-[cd34+/kdr+]EPCs per volume (ml) of whole blood in stroke-prone (sp) female rats that are transgenic Tg25+ (F+) and non-transgenic (F-) compared to non-stroke-prone (nsp) Tg25+ and non-transgenic female rats at two pre-stroke time points: 2 months (2 m) and 4 months (4 m) of age. E-F) Analysis of the effects of age (2 m, 4 m), stroke-susceptibility (sp, nsp) and transgenic hyperlipidemia (F+) vs. nontransgenic normolipidemia (F-) on DEspR+ (E) and DEspR- (F) subclasses of cd45- [cd34+/kdr+]EPCs. Data are presented in box-whiskers plot with minimum/maximum boxed and mean ± s.d. delineated; n = 4–5 rats/study group; EPCs, [cd34+/kdr+] freshly isolated EPCs; *P*-values for one-way ANOVA with Tukey’s all pairwise multiple comparison *, *P*<0.05; **, *P*<0.001, ***, *P*<0.0001, n.s., not significant.

FACS analyses of different EPC parameters were done on whole blood from male and female juvenile 6- and 17 weeks (wk) old rats Dahl S rats. The 17-wk time point was selected to be able to study stroke-prone Tg25 rats prior to strokes, since spTg25 female rats start exhibiting stroke signs around this age [7]. FACS analysis revealed that a typical EPC parameter, the ratio of total [cd34+/kdr+] EPCs to cd45+ white blood cell count, did not exhibit aging-associated changes in both males and females (**Figure 1A**). However, distinguishing the cd45- EPC-subset as the per cent fraction of cd45- [cd34+/kdr+]EPCs in total [cd34+/kdr+]EPCs revealed significant decrease in 17 wk-old males and females, ANOVA *P*<0.0001, (**Figure 1B**) thus indicating informativeness of the cd45- [cd34+/kdr+]EPC subset in this rat stroke model. Concordantly, we also observed that the ratio of cd45+ to cd45- [cd34+/kdr+]EPCs is a robust measure with one way ANOVA *P*<0.0001, and significant post-hoc multiple pairwise comparison (Tukey’s test *P*<0.001) of juvenile and 17 wk-old male and female rats (**Figure 1C**).

### EPC Analysis in Stroke Prone Rats

To specifically test the role of EPCs as juvenile protective factors in the context of aging and stroke susceptibility, we first studied putative changes in EPC subtype-specific numbers in stroke prone rats compared to control non-stroke prone rats that were genetically identical with the exception of developmental programming for stroke induced by gestational exposure to increased NaCl [7]. We selected this stroke-prone rat model since this preclinical stroke model recapitulates stroke risk factors which affect EPC levels and functionality as age, hypertension and hyperlipidemia, and [2]–[6].

Following identical experimental conditions, FACS analysis revealed that juvenile rats had higher levels of cd45- [cd34+/kdr+]EPCs/ml than 4 m-old rats regardless of transgenic expression of human cholesteryl ester transfer protein, which induces significant hypercholesterolemia on regular rat chow [8] (**Figure 1D**). To determine if a new subtyping-scheme of EPCs would give further insight into the dynamic modulation of circulating EPC numbers during cerebrovascular disease progression, we next tested expression of the dual endothelin1/VEGFsp receptor (DEspR, formerly called Dear) on cd45- [cd34+/kdr+]EPCs by 4-label LSRII-FACS analysis. DEspR was selected as a previously untried EPC surface marker detected on some embryonic hemangioblasts and involved in developmental vasculogenesis and angiogenesis leading to embryonic lethality by E10.5–12.5 embryonic day [11]. We observed that DEspR+ and DEspR- cd45- [cd34+/kdr+]EPCs exhibit differential modulation patterns with age, stroke susceptibility and transgenic[hCETP]-induced combined hyperlipidemia (**Figures 1E–1F**). Interestingly, among cd45- [cd34+/kdr+]EPCs, DEspR+ EPCs are increased in spTg25+ female rats at 2-months of age and decreased by 4-months of age (**Figure 1E**) when stroke is imminent at 4.5 months of age [7]. In contrast, DEspR- EPCs are both low at 2-m and 4-m old spTg25+ female rats (**Figure 1F**). In non-stroke prone rats, the levels of juvenile DEspR- EPCs is significantly increased in Tg25+ female rats only.

### EPC Gene Expression Changes with Age

In order to assess whether molecular changes occur in parallel to differential changes in subtype-specific numbers, we analyzed pathway-specific changes in freshly isolated DEspR+ and DEspR- cd45- [cd34+/kdr+]EPCs as modulated by age and stroke susceptibility. Focused pathway-specific analysis was done on representative key genes involved in angiogenesis, cell death, survival, cell adhesion, and stemness. These pathways were selected in order to query gene expression profiles of EPCs pertinent to their roles as progenitor cells needing to survive endothelial stress-conditions, home, repair and maintain endothelial integrity and microvascular networks. We used pathway-specific arrays optimized for nanogram-level RNA analyses in order to directly analyze freshly isolated cd45- [cd34+/kdr+]EPCs subtyped according to DEspR+ or DEspR- expression.

**Figure 2:**
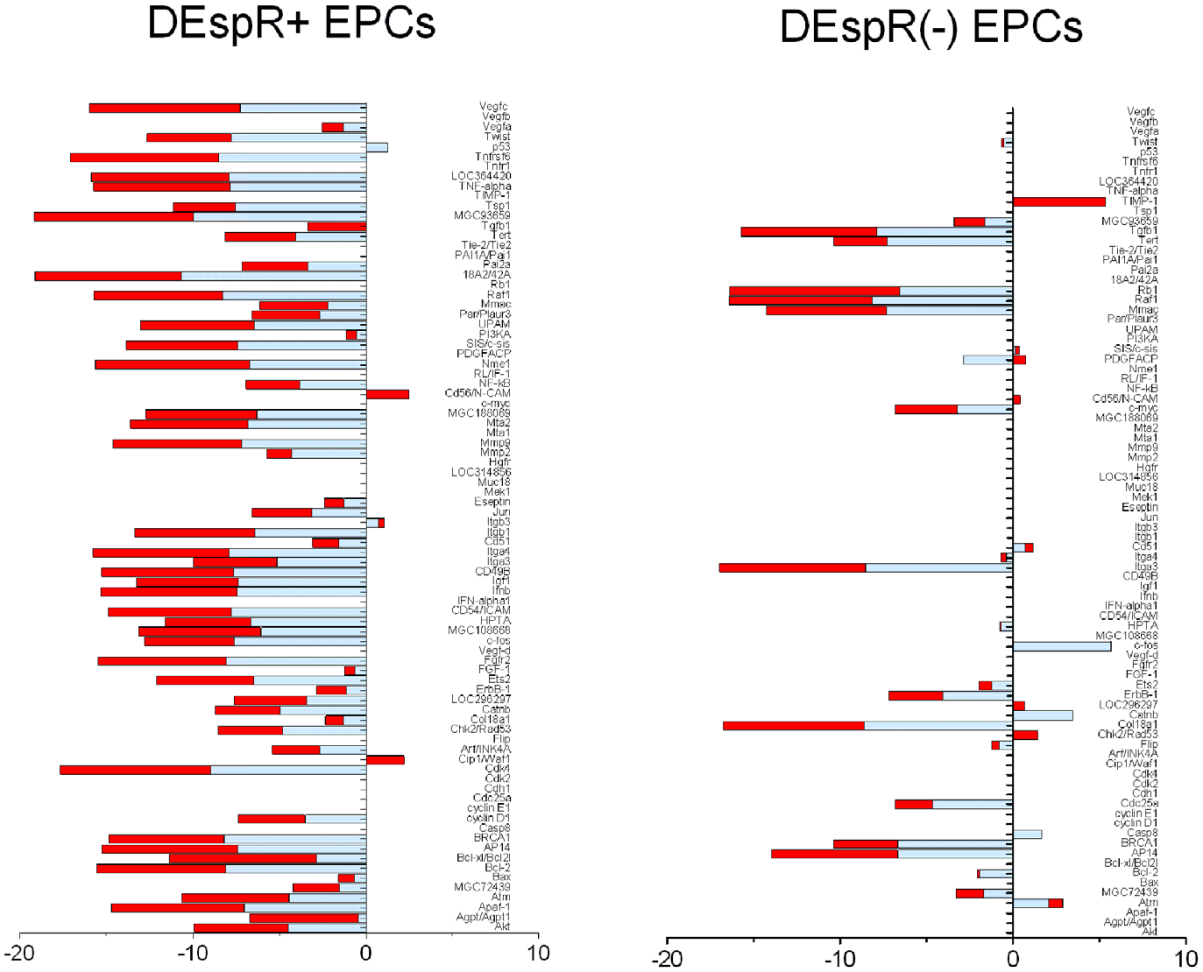
Comparative analysis of impact of age and stroke susceptibility on DEspR+ and DEspR- EPC gene expression profiles. (Specific details in Table S1). Graphical representation of significant gene-specific changes (fold-change: ratio test/common reference) comparing 2 m-old spTg25+ rat EPCs (spY) and non-stroke prone 4 m-old rat EPCs (nspO) reveals similar gene expression changes using 4 m-old spTg25+ rats (spO) as the common reference sample. In both DEspR+ and DEspR- EPCs, most of spY/spO gene expression changes (blue) are similar to nspO/spO gene expression changes (red) with few exceptions. More gene expression changes are noted in DEspR+ EPCs compared with DEspR- EPCs. Significance testing was obtained by 2-way [gene×EPC-subtype] ANOVA followed by Holm-Sidak test for multiple comparisons. Data [gene names, fold change, P-values] presented in Table S1. Negative fold-change, decreased gene expression levels; positive fold-change, increased gene expression levels; zero level, non-expressed genes.

As shown in **Table 1**, significant gene expression changes (P<0.0001) were detected in key pathways contributing to EPC-mediated repair functions: angiogenesis, apoptosis, survival, cell adhesion, and stemness pathways. The following key observations are made. First, age induced differential changes specific to sex and stroke susceptibility. Secondly, stroke susceptibility and age induced the greatest number of subtype-specific gene expression changes in this pathway-specific array. Thirdly, DEspR+ EPCs exhibited more age-associated changes than DEspR- EPCs in stroke prone females and males, but not in non-sp females. Fourth, contrary to predictions for decreased angiogenesis gene expression, age was actually associated with increased angiogenesis-gene expression in stroke-prone female rats except for VEGF which exhibited equivalent levels in all rat groups. Notably, the pro-angiogenic genes induced are also implicated in intimal hyperplasia. Fifth, aging and progression to stroke reduced pro-survival genes in both male and female DEspR+ and DEspR- EPC-subtypes rather than increased apoptosis genes, concordant with decreased EPC numbers and functionality with age. Most importantly, as shown in **Figure 2** and **Table S1**, juvenile (2 m-old) spTg25+ rat EPCs exhibit gene expression profiles similar to old (4 m-old) non-spTg25 rat controls suggesting that accelerated EPC-aging is associated with stroke-susceptibility. This observation is seen in both DEspR+ and DEspR- EPCs, although differential DEspR-subtype specific expression patterns are observed (**Figure 2**).

**Table 1:** Pathway-specific array analysis of cd45- [cd34+/kdr+]EPCs: impact of age, stroke susceptibility and sex.

	Female rats 4 m/2 m: stroke-prone and non-stroke-prone				Non-stroke prone: 6 m/2 m males and 4 m/2 m females				
Pathway & gene name	DEspR+sp-EPCs	DEspR-sp-EPCs	DEspR+nspEPCs	DEspRnspEPCs	DEspR+m-EPCs	DEspRm-EPCs	DEspR+f-EPCs	DEspR-f-EPCs	vasc remodeling
Pro-angiogenesis	>10-x inc*P*-value	>10-x inc*P*-value	>10-x inc*P*-value	>10-x inc*P*-value	>10-x inc*P*-value	>10-x inc*P*-value	>10-x inc*P*-value	>10-x inc*P*-value	Intimal hyperplasia
1. PDGF-β	3×10^−5^	<0.05	x	3×10^−5^	x	x	x	3×10^−5^	rep
2. S100a4	3×10^−5^	<0.03	x	x	x	x	x	x	rep
3. TGFβ-R1	3×10^−5^	3×10^−5^	x	x	x	x	x	x	rep
4. ETS2	3×10^−5^	5×10^−4^	x	x	0.03	x	x	x	
5. FGF-R2	3×10^−5^	X	x	x	x	1×10^−5^	x	x	rep
6. HGF	3×10^−5^	X	x	x	*3×10^−4^	x	x	x	
7. Angp-1	nc	X	x	x	x	x	x	x	
8. IGF-1	3×10^−5^	X	x	x	8×10^−6^	x	x	x	rep
9. MMP-2	7×10^−5^	X	x	x	x	x	x	x	rep
10. MMP-9	3×10^−5^	X	x	x	x	x	x	x	rep
11. Muc1	4×10^−5^	X	x	x	x	x	x	x	
12. Upam	3×10^−5^	X	*5×10^−5^	x	3×10^−4^	x	*5×10^−5^	x	rep
**Apoptosis**									
1. Apaf-1	x	X	x	x	x	x	x	x	
2. Fas	x	X	x	x	x	x	x	x	
3. Caspase 8	x	X	x	nc	x	x	x	nc	
**Survival**									
1. Bcl-2	x	8×10^−5^	x	x	x	8×10^−6^	x	x	
2. BCL-xl	<0.05	X	x	1×10^−3^	<0.05	x	x	1×10^−3^	
3. Survivin	*8×10^−5^	*2×10^−5^	x	x	*8×10^−6^	*2×10^−5^	x	x	
4. BRCA1	*2×10^−5^	X	x	x	*2×10^−5^	x	x	x	
**Cell adhesion**			x	x					
ICAM-1	x	X	x	1×10^−4^	x	x	x	1×10^−4^	rep
**Stemness**									
Twist-1	x	*8×10^−6^	x	x	x	*8×10^−6^	x	x	

Legend: 10-x inc, 10-fold increase in expression level over reference sample; 6 m, 6months; 4 m, 4 months; 2 m, 2 months; EPC, cd45- [cd34+/kdr+] endothelial progenitor cells; DEspR, dual endothelin1/VEGFsp receptor; f, female; m, male; nc, no change; nsp, non-stroke prone, sp, stroke prone; x, not detected, rep, reported involvement in vascular remodeling via intimal hyperplasia; all rat groups with n = 6; *P*-values, from 2-way ANOVA with multiple pairwise comparison, *, decrease in old compared to juvenile sample; inc, increase in old compared to juvenile sample; gene names, GenBank gene names.

### EPCs as Juvenile Protective Factor Therapy

**Figure 3:**
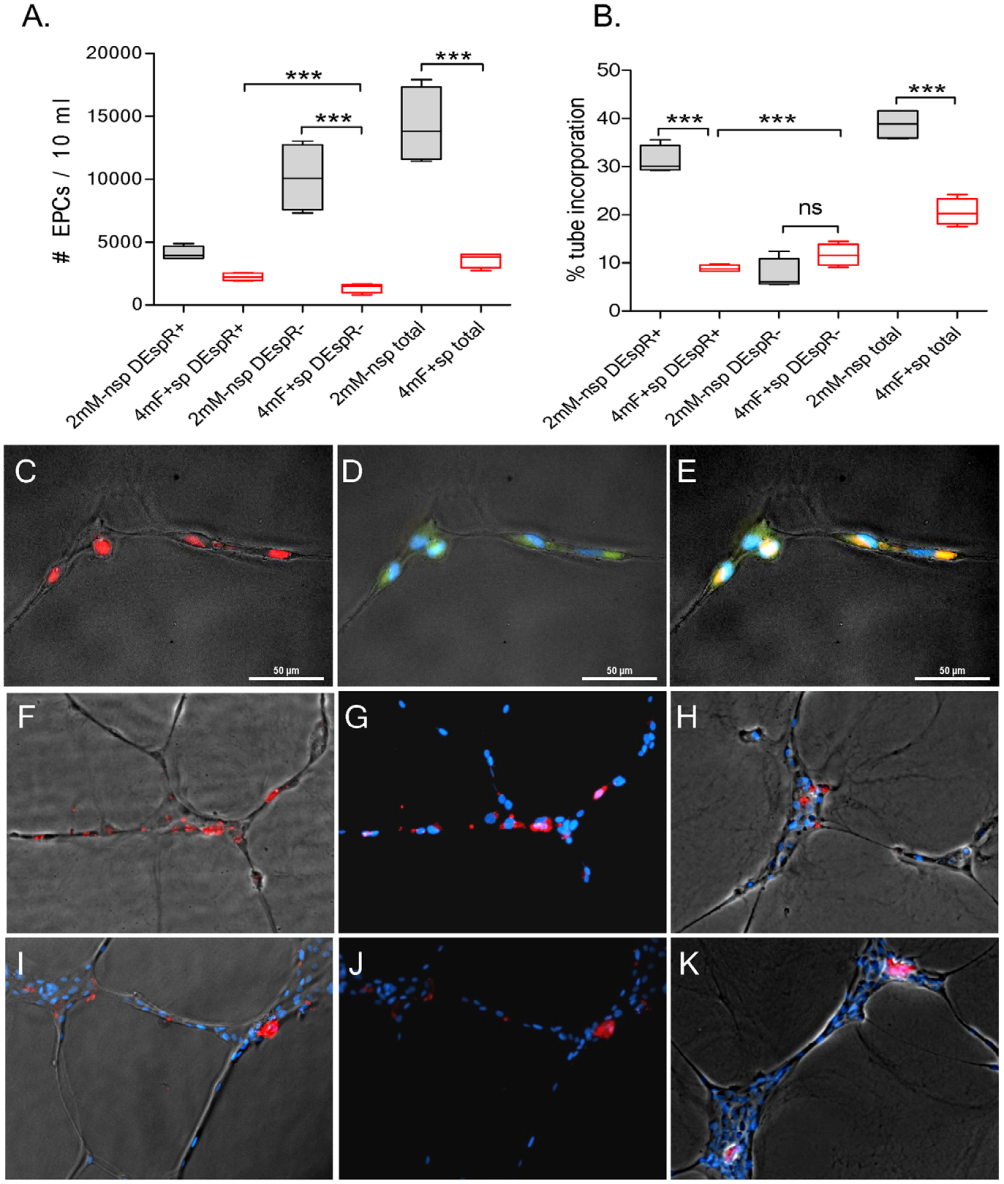
Comparative analysis of EPC numbers and tube-incorporation function. A) Comparison of 4-month old recipient stroke-prone spTg25+ female rats (4 mF+ sp) vs. juvenile donor non-transgenic, non-stroke-prone male rats (2 mM- nsp): number of cd45- [cd34+/kdr+]EPCs per 10 ml of blood subtyped according to DEspR expression (DESpR+, DEspR-). B) Number of DEspR+ and DEspR- cd45- [cd34+/kdr+]EPCs incorporated into angiogenic HUVEC-tubes. C) Representative fluorescent-phase contrast image of pkh26-labeled cd45- [cd34+/kdr+]EPCs (red) incorporated into HUVECs angiogenic tubes. D) DEspR+ (green fluorescence) expression in tube-incorporated EPCs and HUVECs. E) Merged image showing double staining with pkh26 and DEspR+ immunostaining (orange) of tube-incorporated EPCs. F) Representative fluorescent-phase contrast image of pkh26-labeled DEspR+ EPCs isolated from donor juvenile non-stroke prone male showing incorporation into HUVEC tubes. G) Identical image to panel-F without phase-contrast overlay to enhance pkh26-red fluorescence in tubes. H) Representative fluorescent-phase contrast image showing tube incorporation of pkh26-labeled DEspR- EPCs from donor juvenile non-stroke prone male rat. I) Representative fluorescent-phase contrast image of cd45- [cd34+/kdr+]DEspR+ EPCs from recipient 4 m-old spTg25+ female rats that are incorporated into HUVEC tubes. J) Identical image to panel-I without phase contrast overlay. K) Representative fluorescent-phase contrast image of pkh26-labeled cd45- [cd34+/kdr+]DEspR- EPCs incorporated into HUVEC angiogenic tubes. bar = 50 microns; DAPI-stained nuclei (blue); pkh26-fluorescence (red); fluorescence-phase contrast overlay for tube morphology; *P*-values for one-way ANOVA with Tukey’s all pairwise multiple comparison ***, *P*<0.0001, ns, not significant.

**Figure 4:**
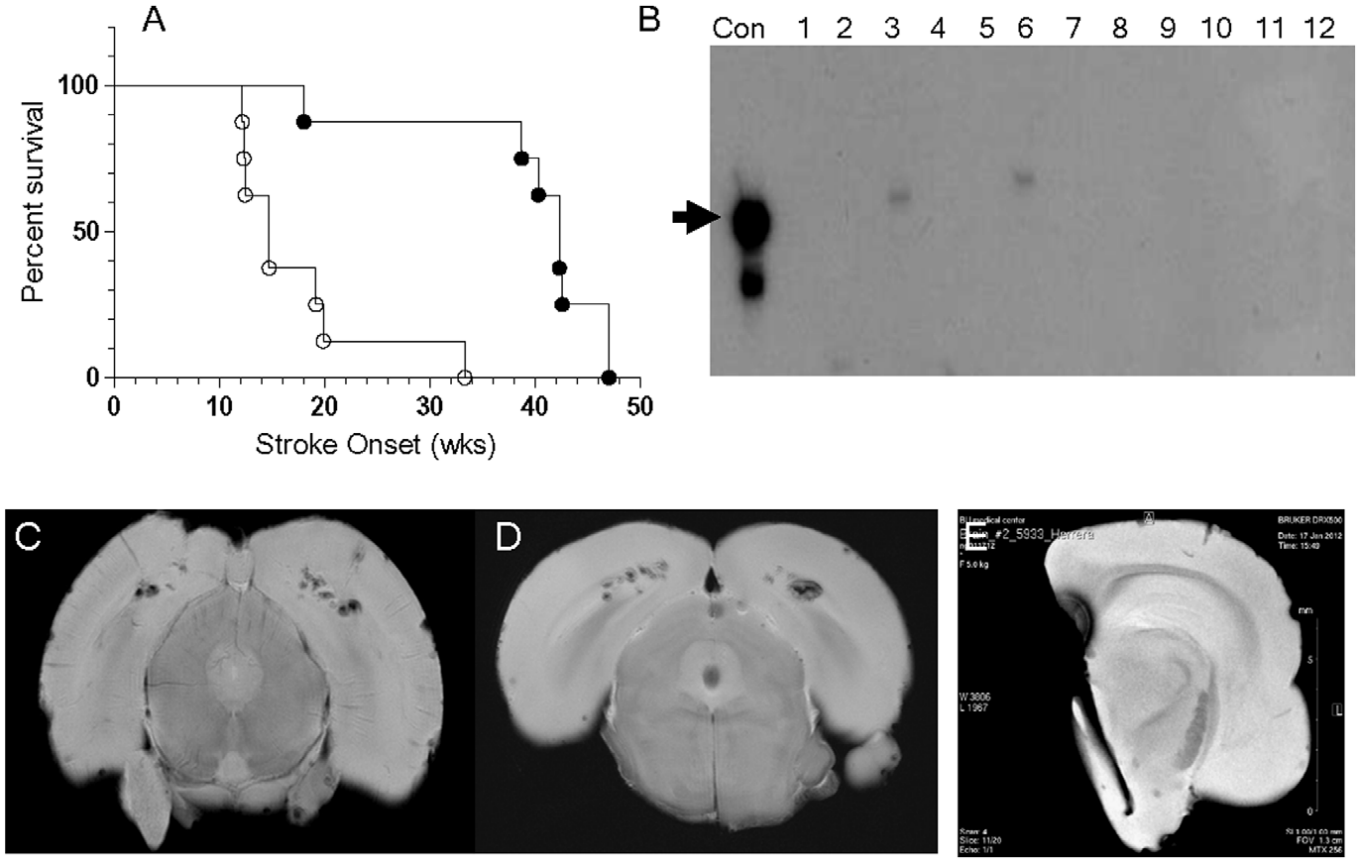
Juvenile EPC-therapy delays stroke onset. A) Survival curve analysis comparing mock-treated controls (○) and EPC-treated (•) spTg25+ female rats, *P* = 0.003. B) PCR-analysis for Y-chromosome DNA detected the expected Y-chromosome sequence-specific 104 bp product in the positive control lane (Con) and in EPC-treated rat brain microvessels at 1 week (lane-3) and 2 weeks (lane-6) after EPC infusion. No Y-DNA was detected at 3- (lane 9) and 4-weeks (lane 12) after EPC-infusion. Y-DNA was not detected in other tissues tested: kidney (lanes 1,4,7,10), and whole brain (lanes 2,5,8,11). C-E) Representative images of gradient-echo MRI analyses of rat brains from: C) a mock-treated rat at stroke onset, D) an EPC-treated rat at significantly-delayed stroke onset, and E) a half-brain from an asymptomatic, EPC-treated rat 3-weeks after EPC-infusion.

To determine translational significance of these changes and prove EPCs as juvenile protective factors, we tested whether infusing juvenile cd45- [cd34+/kdr+]EPCs can delay stroke onset in spTg25+ female rats, known to be the more susceptible to ischemic-hemorrhagic strokes than male rats [7]. Because non-transgenic (nonTg) Dahl Salt-sensitive rat males exhibit the least susceptibility to stroke despite identical developmental programming [7], we assigned them to be the donor syngeneic group so that we can document the integration of male EPCs into brain microvessels by PCR-DNA analysis of Y-chromosome specific markers. Concordant with this donor group selection, analysis of donor juvenile male cd45- [cd34+/kdr+]EPCs by FACS revealed that juvenile male non-stroke prone rats exhibited significantly higher numbers of total cd45- [cd34+/kdr+]EPCs and DEspR- EPCs (Tukey’s all pairwise comparison *P*<0.0001 for both respectively) compared to 4 m stroke-prone spTg25+ female rats (**Figure 3A**). While levels were not different, we next determined whether DEspR+ EPCs might exhibit functional differences in donor males compared to recipient 4 m spTg25+ females, (n = 4–5 rats/group). We tested tube incorporation of pkh26-fluorescently labeled EPCs into HUVECs tube formation in standard pro-angiogenic conditions. We detected that donor juvenile male DEspR+ EPCs exhibited greater tube incorporation capacity compared to recipient 4 m-old spTg25+ female rat DEspR+ EPCs, *P*<0.0001 (Tukey’s all pairwise comparison, **Figure 3B**). We note that both donor male (**Figures 3C, 3D**) and recipient 4 m-old spTg25+ female (**Figures 3E, 3F**) DEspR+ EPCs exhibited tube incorporation functionality, thus eliminating technical confounders. These tube incorporation observations are concordant with observations on EPC function in vivo presented below (**Fig. 4**).

These subtype specific analyses indicated that in order to determine in vivo efficacy of juvenile EPCs as protective factors, infusion of total cd45- [cd34+/kdr+]EPCs makes for the optimal experimental design in order to address both the changes in levels in DEspR- EPCs, and the change in functionality in DEspR+ EPCs. We therefore tested whether infusion of juvenile donor EPCs (from 2 m-old non-stroke prone, nonTg Dahl S male rats) could alleviate the course of microvascular paucity and ensuing chronic low flow ischemia, thereby delaying the onset of ischemic hemorrhagic stroke in the most susceptible subgroup, spTg25+ female rats. Littermate spTg25+ female rats were randomly assigned to EPC therapy at 3- and 4-months of age (n = 8) or mock-therapy infused rats (n = 7). Stroke onset was defined by the onset of neurological deficits such as seizure, paresis/paralysis, or athetoid movements as described [7]. Survival curve analysis of EPC-treated vs. mock-treated rats demonstrated significant delay in the onset of ischemic-hemorrhagic stroke (*P*<0.003) (**Figure 4A**). Retrospective analysis of the number of EPCs infused demonstrates a minimum of 8,500 EPCs/250 gm rat to be effective (**Table 2**).

**Table 2:** Number of juvenile donor non-spTg25- male cd34+/kdr+/cd45- EPCs infused into recipient spTg25+ female rats and corresponding lifespan prior to the onset of stroke.

ID#	Rx-1: EPC #	Rx-2: EPC#	Lifespan (wks)
**1**	**3,985**	**6,099**	**18**
2	14,507	9,720	38.7
3	12,881	10,302	40.29
4	13,498	8,707	42.29
5	14,412	11,388	42.29
6	13,982	18,189	42.57
7	13,372	12,099	47
8	10,520	11,320	47

Legend: Number of total cd34+/kdr+/cd45- EPCs isolated from 2 m-old donor rats (male, non-stroke-prone, nontransgenic, normolipidemic rats) injected into recipient rats (female, stroke prone, transgenic-hyperlipidemic) at 3-months of age (Rx-1) and at 4-months of age (Rx-2). Lifespan is defined by the onset of stroke which prompted euthanasia; wks, weeks of age.

To prove that indeed EPCs are integrated into microvessels, and determine how long donor-EPCs are detectable in recipient rat tissues by PCR-amplification analysis, we performed Y-chromosome DNA analysis on recipient rat tissues after EPC infusions (∼10,000 male donor EPCs) from 1–4 weeks post-infusion. We observed that Y-chromosome DNA was detected only in brain microvessels but not in kidney or whole brain, and only at 1- and 2-weeks post infusion timepoints (**Figure 4B**). To confirm ischemic hemorrhagic strokes, we analyzed rat brains at stroke onset by 11.7 Tesla magnetic resonance imaging (MRI) using gradient echo sequences. Cerebral macrohemorrhages are detected at stroke onset in mock-treated rat brain (**Figure 4C**), and at significantly delayed-stroke onset in EPC-treated rats (**Figure 4D**) confirming the stroke-susceptibility of EPC-treated rats. In contrast, MRI analysis of an EPC-treated rat brain isolated prior to the development of stroke signs revealed the absence of cerebral cortex ischemic-hemorrhagic lesions (**Figure 4E**). These observations indicate that EPC-therapy attenuated the progression to macrohemorrhages in spTg25+ female rats, parallel to the delay in the onset of overt stroke neurological deficits.

## Discussion

Our results support the hypothesis that non-expanded cd45- [cd34+/kdr+]EPCs are juvenile protective factors which change with age, and are capable of attenuating spontaneous ischemic-hemorrhagic stroke progression when infused as freshly isolated juvenile EPCs at ∼4–8×10^4^ EPCs/kg peripheral infusion in older stroke-prone rats prior to the onset of stroke. This is much lower than the 0.5–1.0×10^6^ ex vivo-expanded cd45+ [cd34+/kdr+]EPCs infused in 25g mouse models, or 2–4×10^7^ EPCs/kg [1], [12]. Having used an inbred genetically identical stroke-prone model system and ascertained identical environmental factors, subtype-specific modulation of EPC numbers and function prior to stroke onset can be attributed to age. These age-associated changes in cd45- [cd34+/kdr+]EPC levels and function were accompanied by gene expression changes in angiogenesis and cell-survival pathways. The observed acceleration of non-stroke prone age-associated gene expression changes in 8-week old stroke-prone juvenile rats suggest that the modeling of gestational developmental programming of stroke susceptibility in spTg25+ female rats [7] is associated with epigenetic changes that likely account for gene expression changes in EPCs prior to the onset of stroke risk factors such as hypertension and hypercholesterolemia at 12 weeks of age.

Interestingly, the observed age-associated upregulation of genes implicated in neointimal hyperplasia suggest the pathogenic paradigm that EPC-mediated endothelial repair becomes maladaptive with age and actually induces the neointimal hyperplasia and intimal thickening observed with aging and in cerebrovascular disease [13]. This would be concordant with the observation that age is a major risk factor for neointimal hyperplasia [14]. The convergence of EPC-mediated repair and intimal thickening is also concordant with chronic low flow ischemia and microvascular rarefaction observed in spTg25+ rats [7] given that intimal thickening leads to brain capillary occlusion [15], and could contribute to string capillaries associated with compromised brain microcirculation as seen in Alzheimer’s disease [16]. Additionally, since the queried pathway-specific molecular profile of juvenile stroke-prone spTg25+ rat cd45- [cd34+/kdr+]EPCs is similar to the molecular profile of old non-stroke prone rat cd45- [cd34+/kdr+]EPCs, it becomes apparent that stroke susceptibility induced by developmental programming via early-life Na-exposure [7] accelerates age-associated gene expression changes in spTg25+ rat cd45- [cd34+/kdr+]EPCs in both despr+ despr- subtypes. Modeling this consistently in the spTg2 rat model suggests subtype-specific epigenetic changes in EPCs with age, and that DEspR expression underlies distinct cd45- [cd34+/kdr+] EPC subtypes.

Given that most studies of EPC-therapy are conducted with ex vivo expanded EPCs for cardiac and post-stroke pre-clinical and clinical studies [1], [5], [12], our data showing that freshly isolated juvenile cd45- [cd34+/kdr+]EPCs act as juvenile protective factors for ischemic-hemorrhagic stroke are concordant with studies reporting hematopoietic stem cell roles rather than endothelial repair roles for ex vivo culture-expanded cd45+ EPCs or colony forming cells [17]–[19]. Furthermore, the observation that juvenile EPC-infusions into stroke-prone spTg25+ female rat recipients at 3 m and 4 m of age can delay stroke onset without need for anti-hypertensive therapy suggests a central ‘master-switch’ role for cd45- [cd34+/kdr+]EPCs in stroke pathogenesis via the putative attenuation of microvascular paucity leading to chronic low flow ischemia observed in the spTg25+ female rat model [8]. The detection of donor Y-chromosome DNA in recipient spTg25+ female rat brain microvessels demonstrates that cd45- [cd34+/kdr+]EPCs integrate into brain microvessels sufficient to delay stroke onset in this ischemic-hemorrhagic stroke-prone rat model. The unequivocal detection, albeit low level, of Y-chromosome DNA in recipient spTg25+ female brain microvessels but not in whole brain is not surprising given the number (∼8,000–18,000) of EPCs infused systemically (Table 2). The non-detection of donor-specific Y-chromosome DNA by 3-weeks post-infusion suggests a finite donor-EPC lifespan. Although much more studies need to be done in order to optimize EPC-mediated repair and to determine potential in situ and paracrine repair mechanisms, these observations nevertheless support the hypothesis that cd45- [cd34+/kdr+]EPCs contribute to real-time in vivo endothelial repair and microvascular maintenance, which in the brain would stabilize blood brain barrier integrity and the neurovascular unit. Additionally, these observations are consistent with previous studies reporting that juvenile bone marrow cells restored aging-impaired cardiac angiogenic function [20] and increased microvascular density in 10-month old spontaneously hypertensive female rat brains resulting in reduced peri-infarct size after experimental middle cerebral artery occlusion [21], as well as address aging-induced microcirculatory dysfunction [22].

The detection of 11.7 Tesla MRI evidence of ischemic-hemorrhagic lesions associated with neurologic deficits at stroke onset, and the absence of said lesions when stroke onset is delayed by juvenile EPC cell-therapy suggests that the delay in stroke-onset is likely due to the attenuation of progression to ischemic-hemorrhagic stroke lesions, rather than due to neuroprotection in the presence of ischemic-hemorrhagic lesions. These data suggest that juvenile EPC-therapy likely reduces the microvascular paucity and string-like capillaries observed in spTg25+ female rats [7], and/or stabilizes the blood brain barrier which is disrupted or deficient in post-ischemic stroke [23].

In summary, the data demonstrate that the understudied cd45- [cd34/kdr+]EPC subset comprise juvenile protective factors whose quantitative and qualitative normalization can attenuate the progression of ischemic-hemorrhagic stroke pathogenesis in the spTg25 rat model likely through the maintenance of brain microvascular health. Moreover, the observed age-associated molecular changes in this EPC subset, which involve pro-angiogenic pathways that are also implicated in intimal hyperplasia, link aging EPCs with age-associated neointimal hyperplasia. Most importantly, given no therapy for ischemic-hemorrhagic stroke risk, the identification of cd45- [cd34+/kdr+]EPCs as juvenile protective factors provides key insight into a translational paradigm to attenuate stroke pathogenesis. EPC-therapy for ischemic-hemorrhagic strokes would have pleiotropic effects from the stabilization, if not normalization of the microcirculation, thus potentially promoting neuronal survival and preventing progression of ischemic-hemorrhagic transformation after an acute ischemic event. This projected therapeutic outcome would comply with the deduced requirement for pleiotropic effects for successful stroke therapy [24].

## Supporting Information

Table S1 **Supplementary table of gene names with corresponding fold-change and P-values of pathway-specific array analysis presented pictorially in** **Figure 2** **.**
(DOCX)

## References

[1] Urbich C, Heeschen C, Aicher A, Dernbach E, Zeiher AM, et al (2003) Relevance of monocytic features for neovascularization capacity of circulating endothelial progenitor cells. Circulation 108: 2511–2516. 14581410 10.1161/01.CIR.0000096483.29777.50

[2] Jujo K, Li M, Losordo DW (2008) Endothelial progenitor cells in neovascularization of infarcted myocardium. J Mol Cell Cardiol 45: 530–544. 18755197 10.1016/j.yjmcc.2008.08.003 PMC2628572

[3] Sen S, McDonald SP, Coates PTH, Bonder CS (2011) Endothelial progenitor cells: novel biomarker and promising cell therapy for cardiovascular disease. Clin Science 120: 263–283. 10.1042/CS20100429 21143202

[4] Williamson K, Stringer SE, Alexander MY (2012) Endothelial progenitor cells enter the aging arena. Frontiers in Physiol 3, 1–7. Doi: 10.3389/fphys.2012.00030. 10.3389/fphys.2012.00030 PMC3282536 22363299

[5] Fadini GP, Losordo D, Dimmeler S (2012) Critical reevaluation of endothelial progenitor cell phenotypes for therapeutic and diagnostic use. Circ Res 110: 624–637. 22343557 10.1161/CIRCRESAHA.111.243386 PMC3382070

[6] Navarro-Sobrino M, Rosell A, Hernandez-Guillamon M, Penalba A, Ribo M, et al (2010) Mobilization, endothelial differentiation and functional capacity of endothelial progenitor cells after ischemic stroke. Microvasc Res 80: 317–323. 20594997 10.1016/j.mvr.2010.05.008

[7] Decano JL, Viereck JC, McKee AC, Hamilton JA, Ruiz-Opazo N, et al (2009) Early-life sodium-exposure unmasks susceptibility to stroke in hyperlipidemic, hypertensive heterozygous Tg25 rats transgenic for human cholesteryl ester transfer protein. Circulation 119: 1501–1509. 19273719 10.1161/CIRCULATIONAHA.108.833327 PMC2825876

[8] Herrera VLM, Makrides S, Xie HX, Adari H, Krauss RM, et al (1999) Spontaneous combined hyperlipidemia, coronary heart disease and decreased survival in Dahl salt-sensitive hypertensive rats transgenic for human cholesteryl ester transfer protein. Nat Med 5: 1383–1389. 10581080 10.1038/70956

[9] Bergerat A, Decano JL, Wu CJ, Choi H, Nesvizhskii AI, et al (2011) Prestroke proteomic changes in cerebral microvessels in stroke-prone, transgenic[hCETP]-hyperlipidemic, Dahl salt-sensitive hypertensive rats. Mol Med 17: 588–598. 21519634 10.2119/molmed.2010.00228 PMC3146600

[10] An J, Beauchemin N, Albanese J, Abney TO, Sullivan AK (1997) Use of rat cDNA probe specific for the Y-chromosome to detect male-derived cells. J Andrology 18: 289–293. 9203057

[11] Herrera VLM, Ponce LRB, Bagamasbad P, VanPelt BD, Didishvili T, et al (2005) Embryonic lethality in Dear gene deficient mice: new player in angiogenesis. Physiol Genomics 23: 257–268. 16293765 10.1152/physiolgenomics.00144.2005

[12] Fan Y, Shen F, Frenzel T, Zhu W, Ye J, et al (2010) Endothelial progenitor cell transplantation improves long-term stroke outcome in mice. Ann Neurol 67: 488–497. 20437584 10.1002/ana.21919 PMC3026588

[13] delZopppo GJ (2009) Relationship of neurovascular elements to neuron injury during ischemia. Cerebrovascular Dis. 27:(suppl 1) 65–76. 10.1159/000200442 PMC2914435 19342834

[14] Gennaro G, Menard C, Michaud SE, Rivard A (2003) Age-dependent impairment of reendothelialization after arterial injury. Circulation 107: 230–233. 12538420 10.1161/01.cir.0000050652.47145.4c

[15] Vracko R, Benditt EP (1970) Capillary basal lamina thickening: its relationship to endothelial cell death and replacement. J Cell Biol 47: 281–285. 5513555 10.1083/jcb.47.1.281 PMC2108406

[16] Hunter JM, Kwan J, Malek-Ahmadi M, Maarouf CL, Kokjohn TA, et al (2012) Morphological and pathological evolution of the brain microcirculation in aging and Alzheimer’s disease. PloS ONE 7(5): e36893. 22615835 10.1371/journal.pone.0036893 PMC3353981

[17] Case J, Mead LE, Bessler WK, Prater D, White HA, et al (2007) Human cd34+ac133+vegfr2 cells are not endothelial progenitor cells but distinct, primitive hematopoietic progenitors. Exp Hematol 35: 1109–1118. 17588480 10.1016/j.exphem.2007.04.002

[18] Desai A, Glaser A, Liu D, Raghavachari N, Blum A, et al (2009) Microarray-based characterization of a colony assay used to investigate endothelial progenitor cells and relevance to endothelial function in humans. Arterioscler Thromb Vasc Biol 29: 121–127. 19092138 10.1161/ATVBAHA.108.174573 PMC2664554

[19] Case J, Mead LE, Bessler WK, Prater D, White HA, et al (2007) Human cd34+ac133+vegfr2+ cells are not endothelial progenitor cells but distinct, primitive hematopoietic progenitors. Exp Hematology 35: 1109–1118. 10.1016/j.exphem.2007.04.002 17588480

[20] Edelberg JM, Tang L, Hattori K, Lyden D, Rafii S (2002) Young adult bone marrow-derived endothelial precursor cells restore aging-impaired cardiac angiogenic function. Circ Res 90: e89–e93. 12039806 10.1161/01.res.0000020861.20064.7e

[21] Taguchi A, Zhu P, Cao F, Kikuchi-Taura A, Kasahara Y, et al (2011) Reduced ischemic brain injury by partial rejuvenation of bone marrow cells in aged rats. J Cereb Blood Flow Metab 31: 855–867. 20859292 10.1038/jcbfm.2010.165 PMC3063619

[22] Payne GW, Bearden SE (2006) The microcirculation of skeletal muscle in aging. Microcirculation 13: 275–277. 16611593 10.1080/10739680600618710

[23] Borlongan CV (2011) Bone marrow stem cell mobilization in stroke: a ‘bonehead’ may be good after all! Leukemia. 25: 1674–1686. 10.1038/leu.2011.167 PMC3291108 21727900

[24] Woodruff TM, Thundyil J, Tang SC, Sobey CG, Taylor SM, et al (2011) Pathophysiology, treatment, and animal and cellular models of human ischemic stroke. Molecular Neurodegeneration 6 11: 1–19. 10.1186/1750-1326-6-11 PMC3037909 21266064

