# Supplementary material for: Analysis of cd45- [cd34+/kdr+] Endothelial Progenitor Cells as Juvenile Protective Factors in a Rat Model of Ischemic-Hemorrhagic Stroke
Source: PLoS One. 2013 Jan 31;8(1):e55222. doi: 10.1371/journal.pone.0055222 (PMC3561358; doi:10.1371/journal.pone.0055222)
Supplement: Table S1 — Supplementary table of gene names with corresponding fold-change and P-values of pathway-specific array analysis presented pictorially in Figure 2 . (DOCX) [file pone.0055222.s001.docx]

**Supplementary Table S1.**

|  |  | **DEspR(+)** cd45- [cd34+/kdr+]EPCs | | | | |  | **DEspR(-)** cd45- [cd34+/kdr+]EPCs | | | | |
| --- | --- | --- | --- | --- | --- | --- | --- | --- | --- | --- | --- | --- |
|  |  |  |  |  |  |  |  |  |  |  |  |  |
| Gene name |  | spY / spO | *P* |  | nspO/spO | *P* |  | spY / spO | *P* |  | nspO / spO | *P* |
| Akt |  | 0.04 | 0.0007 |  | 0.02 | 0.00003 |  |  |  |  |  |  |
| Agpt/Agpt1 |  | 0.74 | ns |  | 0.01 | 0.00002 |  |  |  |  |  |  |
| Apaf-1 |  | 0.01 | 0.00002 |  | 0.005 | 0.00002 |  |  |  |  |  |  |
| Atm |  | 0.05 | 0.0001 |  | 0.01 | 0.00002 |  |  |  |  |  |  |
| Bax |  | 0.63 | ns |  | 0.53 | ns |  | 0.31 | ns |  | 0.34 | ns |
| Bcl-2 |  | 0.004 | 0.00002 |  | 0.01 | 0.00002 |  |  |  |  |  |  |
| Bcl-xl/Bcl2l |  | 0.14 | ns |  | 0.003 | 0.00002 |  | 0.26 | ns |  | 0.94 | ns |
| AP14 |  | 0.01 | 0.00002 |  | 0.004 | 0.00002 |  |  |  |  |  |  |
| BRCA1 |  | 0.003 | 0.00002 |  | 0.01 | 0.00002 |  | 0.01 | 0.00002 |  | 0.01 | 0.00002 |
| Casp8 |  |  |  |  |  |  |  | 0.01 | 0.00007 |  | 0.08 | 0.0505 |
| cyclin D1 |  | 0.09 | 0.037 |  | 0.07 | 0.009 |  | 3.18 | ns |  |  |  |
| Cdh1 |  |  |  |  |  |  |  | 0.04 | 0.0007 |  | 0.22 | ns |
| Cdk4 |  | 0.002 | 0.00002 |  | 0.002 | 0.00002 |  |  |  |  |  |  |
| Cip1/Waf1 |  |  |  |  | 4.69 | 0.077 |  |  |  |  |  |  |
| Arf/INK4A |  | 0.16 | ns |  | 0.15 | ns |  |  |  |  |  |  |
| Chk2/Rad53 |  | 0.04 | 0.002 |  | 0.07 | 0.03 |  | 0.58 | ns |  | 0.73 | ns |
| Col18a1 |  | 0.40 | ns |  | 0.49 | ns |  |  |  |  | 2.75 | ns |
| Catnb |  | 0.03 | 0.0005 |  | 0.07 | 0.008 |  | 0.003 | 0.00002 |  | 0.003 | 0.00002 |
| LOC296297 |  | 0.09 | ns |  | 0.05 | 0.012 |  | 11.05 | 0.0004 |  |  |  |
| ErbB-1 |  | 0.45 | ns |  | 0.31 | ns |  | 0.97 | ns |  | 1.59 | ns |
| Ets2 |  | 0.01 | 0.00002 |  | 0.02 | 0.00002 |  | 0.06 | 0.0001 |  | 0.12 | 0.004 |
| FGF-1 |  | 0.66 | ns |  | 0.66 | ns |  | 0.43 | ns |  | 0.60 | ns |
| Fgfr2 |  | 0.004 | 0.00002 |  | 0.01 | 0.00002 |  |  |  |  |  |  |
| c-fos |  | 0.005 | 0.00002 |  | 0.03 | 0.0002 |  |  |  |  |  |  |
| MGC108668 |  | 0.01 | 0.0003 |  | 0.01 | 0.00002 |  | 50.87 | 0.00002 |  |  |  |
| HPTA |  | 0.01 | 0.00002 |  | 0.03 | 0.001 |  |  |  |  |  |  |
| CD54/ICAM |  | 0.005 | 0.00002 |  | 0.01 | 0.00002 |  | 0.62 | ns |  | 0.94 | ns |
| IFN-alpha1 |  |  |  |  |  |  |  |  |  |  |  |  |
| Ifnb |  | 0.01 | 0.00002 |  | 0.004 | 0.00002 |  |  |  |  |  |  |
| Igf1 |  | 0.01 | 0.00002 |  | 0.02 | 0.00002 |  |  |  |  |  |  |
| CD49B |  | 0.005 | 0.00002 |  | 0.005 | 0.00002 |  |  |  |  |  |  |
| Itga3 |  | 0.03 | 0.0003 |  | 0.03 | 0.0009 |  |  |  |  |  |  |
| Itga4 |  | 0.004 | 0.00002 |  | 0.004 | 0.00002 |  | 0.003 | 0.00002 |  | 0.003 | 0.00002 |
| Cd51 |  | 0.33 | ns |  | 0.36 | ns |  | 0.77 | ns |  | 0.81 | ns |
| Itgb1 |  | 0.01 | 0.00002 |  | 0.01 | 0.00002 |  | 1.62 | ns |  | 1.39 | ns |
| Itgb3 |  | 1.63 | ns |  | 1.29 | ns |  |  |  |  |  |  |
| Jun |  | 0.11 | 0.003 |  | 0.09 | 0.003 |  |  |  |  |  |  |
| Mmp2 |  | 0.05 | 0.00003 |  | 0.37 | ns |  |  |  |  |  |  |
| Mmp9 |  | 0.01 | 0.00002 |  | 0.01 | 0.00002 |  |  |  |  |  |  |
| Mta1 |  |  |  |  |  |  |  |  |  |  |  |  |
| Mta2 |  | 0.01 | 0.00002 |  | 0.01 | 0.00002 |  |  |  |  |  |  |
| MGC188069 |  | 0.01 | 0.00002 |  | 0.01 | 0.00002 |  |  |  |  |  |  |
| Cd56/N-CAM |  |  |  |  | 5.69 | ns |  | 0.11 | 0.019 |  | 0.08 | 0.012 |
| NF-kB |  | 0.07 | 0.015 |  | 0.11 | ns |  | 1.03 | ns |  | 1.31 | ns |
| Nme1 |  | 0.01 | 0.00005 |  | 0.002 | 0.00002 |  |  |  |  |  |  |
| SIS/c-sis |  | 0.01 | 0.00002 |  | 0.01 | 0.00002 |  | 0.14 | 0.055 |  | 1.68 | ns |
| PI3KA |  | 0.70 | ns |  | 0.65 | ns |  | 1.08 | ns |  | 1.18 | ns |
| UPAM |  | 0.01 | 0.00002 |  | 0.01 | 0.00002 |  |  |  |  |  |  |
| Par/Plaur3 |  | 0.16 | ns |  | 0.07 | 0.006 |  |  |  |  |  |  |
| Mmac |  | 0.22 | ns |  | 0.06 | 0.005 |  |  |  |  |  |  |
| Raf1 |  | 0.003 | 0.00002 |  | 0.01 | 0.00002 |  | 0.01 | 0.00002 |  | 0.01 | 0.00002 |
| Rb1 |  |  |  |  |  |  |  | 0.004 | 0.00002 |  | 0.003 | 0.00002 |
| 18A2/42A |  | 0.001 | 0.00002 |  | 0.003 | 0.00002 |  | 0.01 | 0.026 |  | 0.001 | 0.00002 |
| Pai2a |  | 0.10 | 0.0003 |  | 0.07 | 0.00008 |  |  |  |  |  |  |
| Tert |  | 0.06 | 0.029 |  | 0.06 | 0.019 |  |  |  |  |  |  |
| Tgfb1 |  |  |  |  | 0.10 | 0.003 |  | 0.006 | 0.00002 |  | 0.12 | ns |
| Tsp1 |  | 0.005 | 0.00002 |  | 0.08 | 0.008 |  | 0.32 | ns |  | 0.30 | ns |
| TNF-alpha |  | 0.004 | 0.00002 |  | 0.004 | 0.00002 |  |  |  |  | 41.63 | 0.00002 |
| Tnfrsf6 |  | 0.003 | 0.00002 |  | 0.003 | 0.00002 |  |  |  |  |  |  |
| Twist |  | 0.005 | 0.00002 |  | 0.03 | 0.002 |  |  |  |  |  |  |
| Vegfa |  | 0.41 | ns |  | 0.43 | ns |  | 0.70 | ns |  | 0.91 | ns |
| Vegfc |  | 0.01 | 0.00002 |  | 0.002 | 0.00002 |  |  |  |  |  |  |
